# Supplementary material for: Single-trajectory map equation
Source: Sci Rep. 2023 Apr 22;13:6597. doi: 10.1038/s41598-023-33880-y (PMC10122677; doi:10.1038/s41598-023-33880-y)
Supplement: Supplementary file 1 — Supplementary Information. [file 41598_2023_33880_MOESM1_ESM.pdf]

Supplementary Materials

"Single-trajectory map equation"

Tatsuro Kawamoto

S1. SUMMARY OF THE AVERAGE CODE LENGTHS

In Table I, we show a summary table of the average code lengths considered in this study.

TABLE I. Symbols and descriptions for the average code lengths considered in the map equation and the single-trajectory map equation.

Symbol	Description
$\mathcal{L}\left(\boldsymbol{\zeta}, \boldsymbol{\sigma}\right)$	Average code length of trajectory $\boldsymbol{\zeta}$ with node partition $\boldsymbol{\sigma}$
$\overline{\mathcal{L}}_{\lambda}\left(\boldsymbol{\sigma}; \{\boldsymbol{\zeta}_a\}\right)$	• Average code length of trajectories $\{\boldsymbol{\zeta}_a\}$ with node partition $\boldsymbol{\sigma}$<br>and hyperparameter λ<br>• An objective function of the single-trajectory map equation<br>• $\overline{\mathcal{L}}\left(\boldsymbol{\sigma}; \{\boldsymbol{\zeta}_a\}\right) = \overline{\mathcal{L}}_{\lambda=1}\left(\boldsymbol{\sigma}; \{\boldsymbol{\zeta}_a\}\right)$
$\underline{\mathcal{L}}_{\lambda}\left(\boldsymbol{\sigma}; \{\boldsymbol{\zeta}_a\}\right)$	• Lower bound of the average code length of trajectories $\{\boldsymbol{\zeta}_a\}$<br>with node partition $\boldsymbol{\sigma}$ and hyperparameter λ<br>• An objective function of the single-trajectory map equation<br>• $\underline{\mathcal{L}}\left(\boldsymbol{\sigma}; \{\boldsymbol{\zeta}_a\}\right) = \underline{\mathcal{L}}_{\lambda=1}\left(\boldsymbol{\sigma}; \{\boldsymbol{\zeta}_a\}\right)$
$L\left(\boldsymbol{\sigma}\right)$	(`--two-level` option in Infomap)<br>• Expected average code length of the random walk with node partition $\boldsymbol{\sigma}$<br>• An objective function of the map equation that is mainly considered<br>in the original paper
$L\left(\boldsymbol{\sigma}\right)$	(`--flow-model rawdir` option in Infomap)<br>• Expected average code length of the flow based on the set of transition<br>probabilities induced by the edges under node partition $\boldsymbol{\sigma}$<br>• An objective function of the map equation that is implemented in Infomap<br>as a variant

S2. ACCURACY OF INFOMAP+ ON THE SBM

For the experiment conducted on the SBM in the main text, we examined whether Infomap+ can correctly estimate the planted number of modules. Readers might have doubts whtether the partitions obtained by Infomap+ are consistent with the planted module structure even when the number of modules is accurately estimated. To clarify this point, we conducted the same experiment on the SBM and measured the fraction of the correctly classified nodes, which is defined as

$$\frac{1}{N}\max\left\{\sum_{i=1}^{N}\delta_{\sigma_i,\sigma_i^*},\sum_{i=1}^{N}(1-\delta_{\sigma_i,\sigma_i^*})\right\}, \tag{S1}$$

where $\sigma_i \in \{1,2\}$ is the inferred module label and $\sigma_i^* \in \{1,2\}$ is the planted module label. Note that $\sum_{i=1}^{N}(1-\delta_{\sigma_i,\sigma_i^*})/N = 1$ indicates that the algorithm perfectly inferred the planted module structure, but with the opposite module label for each node. The value of Eq. (S1) ranges from 0.5 to 1. Figure 1 shows that, when Infomap+ correctly estimates the planted number of modules ($K = 2$), the fraction of correctly classified nodes is indeed high.

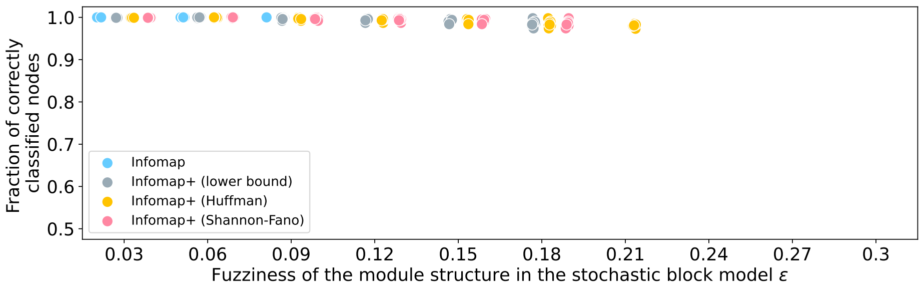

FIG. 1. Fraction of correctly classified nodes by Infomap and Infomap+ in networks generated using the symmetric SBM ($N = 1,000$, $c = 12$). We generated 10 instances of the SBM for each fuzziness of the module structure ϵ and omitted the partitions with $K \neq 2$. Again, we set $\lambda = 1$.

S3. DETAILS OF THE EXPERIMENTS ON REAL-WORLD NETWORK DATASETS

This section describes the details of the real-world networks analysed in the main text and the settings of the algorithms applied. Table II shows the types, number of nodes, and number of edges of the datasets (see the references for the description of each dataset). Table II also lists the value of the hyperparameter λ used in the single-trajectory map equation. Although the Les Miserables network was originally distributed as a weighted network, we converted it to a network with multiple edges because the edge weight represents the number of scene coappearances of characters.

Recall that a large value of λ penalises the generation of new modules. Therefore, starting with $\lambda = 1$, we increased the value of λ little by little (here, with a step of 0.1) until the size of the smallest module became sufficiently large. As far as we have investigated, $\lambda = 1$ is often already sufficient.

As the option in Infomap, we used `--two-level` for the undirected networks and `--two-level, --directed` for the directed networks (these are the methods introduced in the original paper on the map equation). The comparative analysis is fairer and more nontrivial with these options than the experiments with the `--flow-model rawdir` option because the distinction between the flow-based method and trajectory-based method becomes more prominent. We employed the `--two-level` constraint because the evaluation of multilevel partitioning is beyond the scope of the present study.

We can obtain a smaller-sized module or a smaller number of modules using Infomap+ than when using Infomap. This is because Infomap returns different results with different options (recall that we use `--flow-model rawdir` for the initialisation in Infomap+) or different runs.

S4. BIKE-SHARING DATASET

The bike-sharing dataset analysed in the main text was constructed using the dataset distributed through [S11]. The original dataset consists of riding records of a bike-sharing service in London during 2014; for each use (travel),

TABLE II. Real-world network datasets analysed in the main text and the value of the hyperparameter λ (for the lower bound, Huffman coding, and Shannon-Fano coding) used in the single-trajectory map equation.

Dataset name	Type	Nodes	Edges	References	λ
karate club	undirected, simple graph	34	78	[S1, S2]	1, 1, 1
Les Miserables	undirected, multigraph	77	254	[S2]	1, 1, 1
political books	undirected, simple graph	105	441	[S2, S3]	1, 1, 1
football	undirected, simple graph	115	613	[S2]	1, 1, 1
C-elegans-frontal	directed, simple graph	131	764	[S4, S5]	1, 1, 1
E. coli transcription	directed, simple graph	328	456	[S6] (v1.1)	1.3, 1.2, 1.1
email-Eu-core	directed, simple graph	986	25,552	[S5, S7]	1, 1, 1
political blogs	directed, simple graph	1,222	33,428	[S8, S9]	1, 1, 1
power grid	undirected, simple graph	4,941	6,594	[S2]	1.6, 1.7, 1.6
wiki-Vote	directed, simple graph	7,066	103,663	[S5, S10]	1, 1, 1

we can retrieve the starting and ending stations, the time that the bike is used, and the bike ID. Because we have a record of bike IDs, we can track the sequence of the stations that an individual bike has visited. Although the dataset in [S11] is already a subset of larger raw data, we further conducted filtering based on the following criteria:

- We consider the uses on July 31, 2014.
- We focus on the bikes that visited any of the 50 stations that are used most frequently.
- We only consider the series of uses in which the ending station of the previous use coincides with the starting station of the subsequent use.
- We exclude the uses in which the starting and ending stations are identical.

Consequently, we obtained a total of 5,423 trajectories. We used the `--two-level --directed` option to run Infomap.

S5. COMPARISON WITH OTHER VARIANTS OF THE MAP EQUATION

A. Performance of Infomap with the Markov-time parameter

Figures 2 and 3 show the performance of Infomap with the Markov-time parameter (which we refer to as τ) on the SBM and real-world networks, corresponding to the experiments performed in the main text. Note that there is no nontrivial default value of τ; $\tau = 1$ corresponds to the standard Infomap, and we must choose a value $\tau > 1$ to obtain a result distinct from that without the Markov-time parameter. Therefore, we raised τ from 1 little by little (with a step of 0.1) until the size of the smallest module was not less than $\max\{3, N/100\}$; when this condition was not satisfied, we set $\tau = 100$.

In some cases, this approach also provides a partition similar to that based on the single-trajectory map equation. However, because the Markov-time parameter approach globally modifies the resolution scale of partitions, large modules are often merged together as well, at the expense of pruning small modules. For example, in the experiment conducted on the SBM, the performance is considerably deteriorated in terms of the detectability limit when the Markov-time parameter is large; that is, the planted modules are merged even when the fuzziness parameter ϵ is small. Note also that the range of τ we need to sweep is relatively large. By contrast, in the single-trajectory map equation, $\lambda = 1$ or a slightly larger value is often sufficient to avoid overfitting.

B. Performance of the Bayesian Infomap

Figures 4 and 5 show the performance of the Bayesian Infomap [S12, S13] on the SBM and real-world networks, corresponding to the experiments performed in the main text. The Bayesian Infomap has a hyperparameter $\tilde{\lambda}$ that specifies the strength of the prior distribution based on a random network. The default value is $\tilde{\lambda} = (\ln N)/N$; in Infomap, the parameter `regularisation_strength` controls the coefficient a in $\tilde{\lambda} = a(\ln N)/N$.

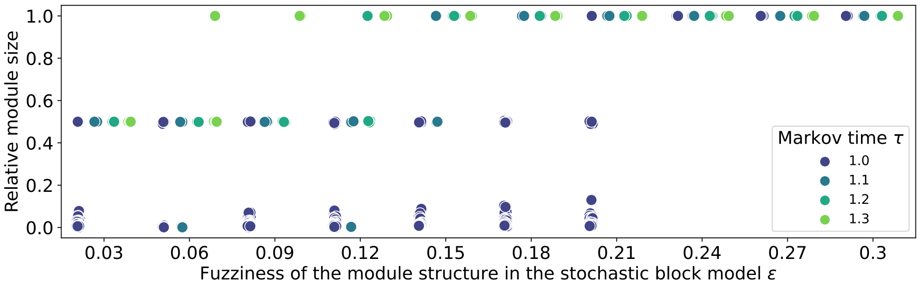

FIG. 2. Performance of Infomap with different values of the Markov-time parameter on the symmetric SBM ($N = 1{,}000$, $c = 12$). We generated five instances of the SBM for each fuzziness of the module structure ϵ and plotted the distribution of the resulting relative module sizes.

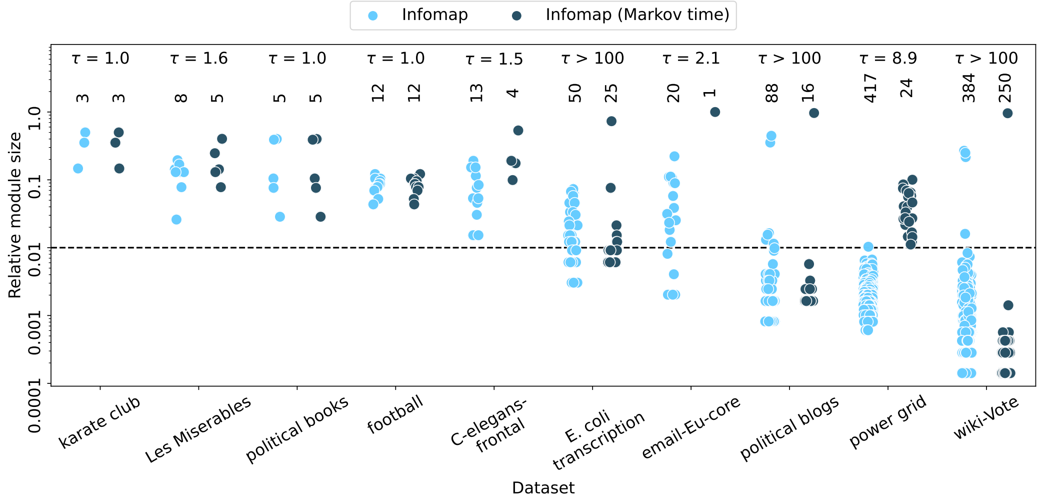

FIG. 3. Relative module sizes obtained by Infomap (without the Markov-time parameter) and Infomap with different values of the Markov-time parameters on the real-world networks considered in the main text. The number of identified modules and the value of the Markov-time parameter τ are depicted at the top of each result. We selected the minimum value of τ such that the smallest module is not less than $\max\{3, N/100\}$ (the dashed line represents 0.01); otherwise, we set $\tau = 100$ (and denote "$\tau > 100$").

Overall, the Bayesian Infomap is highly sensitive to the choice of $\tilde{\lambda}$. As observed in Fig. 4, in many cases, the Bayesian Infomap either leaves many small modules or identifies the whole network as a module. The same tendency was observed for the real-world networks, as shown in Fig. 5. Similar to the experiment in the main text, we increased the value of $\tilde{\lambda}$ from zero (with a step of 0.1) until the size of the smallest module was not less than $\max\{3, N/100\}$. As a result, the Bayesian Infomap did not identify nontrivial modules for the large networks. We also conducted a version in which the threshold of the smallest module was $\max\{3, N/1{,}000\}$. However, the same number of modules was obtained for each dataset.

In summary, although the Bayesian Infomap also aims to avoid overfitting, its performance is distinct from that of the single-trajectory map equation. For the datasets we have investigated, it was not easy to prune small modules while continuing to identify large modules. However, it should also be noted that the Bayesian Infomap is a highly flexible method that the performance can be improved by tuning the prior distribution more carefully.

[S1] W. W. Zachary, J. Anthropol. Res. **33**, 452 (1977).

[S2] `http://www-personal.umich.edu/~mejn/netdata/`.

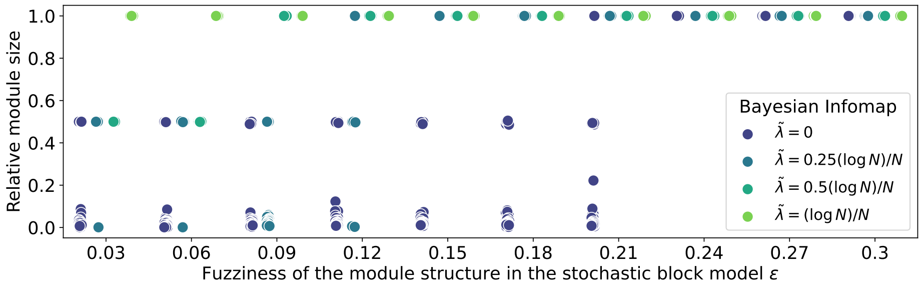

FIG. 4. Performance of the Bayesian Infomap with different values of the prior parameter $\tilde{\lambda}$ on the symmetric SBM ($N = 1,000$, $c = 12$). We generated five instances of the SBM for each fuzziness of the module structure ϵ and plotted the distribution of the resulting relative module sizes.

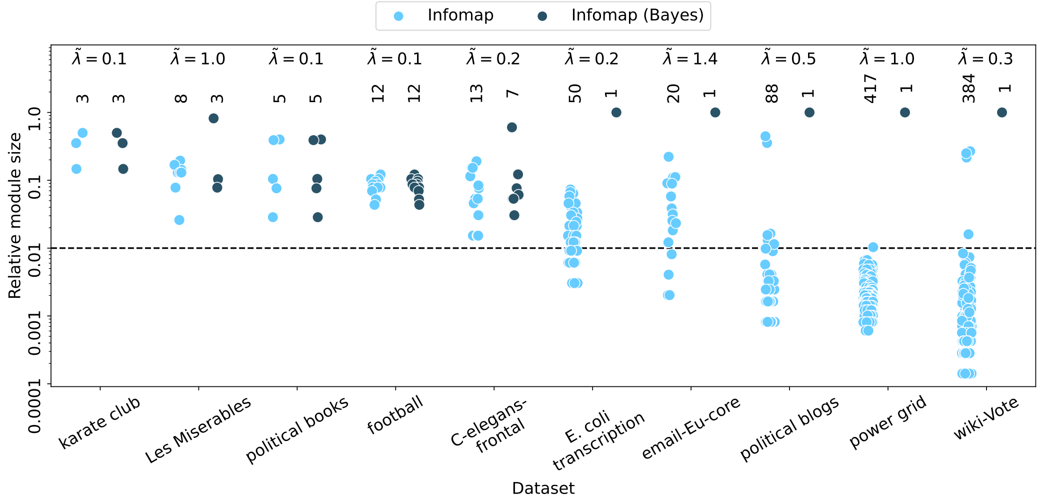

FIG. 5. Relative module sizes obtained by Infomap (without the Bayesian regularisation) and the Bayesian Infomap with different values of the prior parameter for the real-world networks considered in the main text. The number of identified modules and the value of the prior parameter $\tilde{\lambda}$ are depicted at the top of each result. We selected the minimum value of $\tilde{\lambda}$ such that the smallest module is not less than $\max\{3, N/100\}$ (the dashed line represents 0.01).

[S3] M. E. J. Newman, Proc. Natl. Acad. Sci. U.S.A. **103**, 8577 (2006).

[S4] M. Kaiser and C. C. Hilgetag, PLOS Computational Biology **2**, 1 (2006).

[S5] `http://konect.cc/networks/`.

[S6] `https://www.weizmann.ac.il/mcb/UriAlon/e-coli-transcription-network`.

[S7] H. Yin, A. R. Benson, J. Leskovec, and D. F. Gleich, in *Proceedings of the 23rd ACM SIGKDD international conference on knowledge discovery and data mining* (2017) pp. 555–564.

[S8] L. A. Adamic and N. Glance, in *Proceedings of the 3rd International Workshop on Link Discovery*, LinkKDD '05 (ACM, New York, NY, USA, 2005) pp. 36–43.

[S9] `https://snap.stanford.edu/data/`.

[S10] J. Leskovec, D. Huttenlocher, and J. Kleinberg, in *Proceedings of the 19th International Conference on World Wide Web*, WWW '10 (Association for Computing Machinery, New York, NY, USA, 2010) p. 641–650.

[S11] `https://github.com/konstantinklemmer/bikecommclust`.

[S12] J. Smiljanić, D. Edler, and M. Rosvall, Phys. Rev. E **102**, 012302 (2020).

[S13] J. Smiljanić, C. Blöcker, D. Edler, and M. Rosvall, J. Comp. Net. **9** (2021).

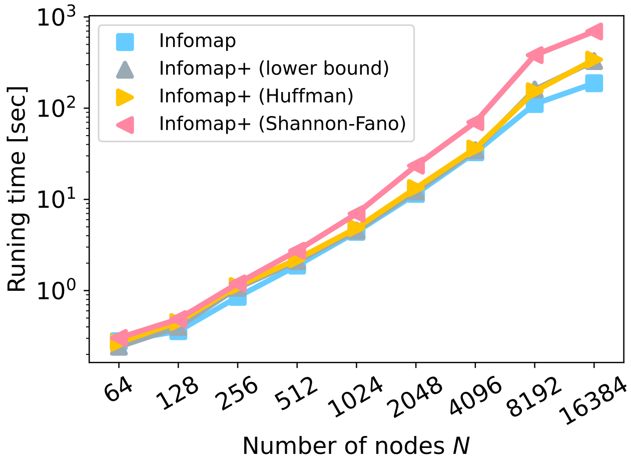

FIG. 6. Running times of Infomap (`--two-level`) and Infomap+ on the SBM. Each point represents the mean running time for five network instances generated from the SBM with eight equally-sized planted modules ($c = 12$, $\epsilon = 0.1$). The running time of each algorithm grows polynomially with N.
